# Nogier Reflex: Physiological and Experimental Results in Auricular Medicine–A New Hypothesis

**DOI:** 10.3390/medicines5040132

**Published:** 2018-12-12

**Authors:** Gerhard Litscher, Theodoros Yannacopoulos, Peter Kreisl

**Affiliations:** 1 Research Unit for Complementary and Integrative Laser Medicine, Research Unit of Biomedical Engineering in Anesthesia and Intensive Care Medicine, and TCM Research Center Graz, Medical University of Graz, Auenbruggerplatz 39, EG19, 8036 Graz, Austria; 2 Acupunktur Zurich GmbH, Seefeldstrasse 283, 8008 Zurich, Switzerland; info@acupunktur.ch; 3 Biochemist, Gerberweg 7, 24211 Preetz, Germany; kreisl@web.de

**Keywords:** auricular medicine, auricular therapy, Nogier reflex, reflex auriculo-cardiac (RAC), vascular autonomic signal (VAS), new hypothesis, ear acupuncture, ear stimulation

## Abstract

This editorial describes a new hypothesis concerning the nature and possible mechanisms of the Nogier reflex or reflex auriculo-cardiac (RAC; also vascular autonomic signal VAS). A multimodal concept for future RAC research is proposed.

At the moment there is no general scientific consensus on the topic concerning Nogier reflex or reflex auriculo-cardiac (RAC; also vascular autonomic signal VAS). However, nevertheless it is an important method in auricular medicine. This editorial introduces a new hypothesis and an approach for the explanation and detection of RAC. The first evidence-based data and some pilot measurements are presented here.

Based on an electronic detection of the so-called RAC pulse reflex according to Paul Nogier [1,2], the description presented here is intended to assist either inexperienced therapists by means of optical, acoustic, electric or other stimuli signaling in order to make the signal recordable. However, in the future maybe it can also be used for controlling bioresonance devices in the sense of a biofeedback function.

The so-called RAC phenomenon was first described by Paul Nogier in the 1970s [1,2,3]. Based on the assumption that most cells of the organism respond to stimuli of the autonomic nervous system, Nogier sought a method that allowed the body’s response to acupuncture-induced measures to be presented. He discovered that by mechanical pressure on certain points on the auricle in his opinion, a cardiovascular reaction took place. He called this reaction “réflexe auriculo cardiaque”, later also abbreviated as RAC [4].

The research of Frank Bahr offered RAC for its practical application in the field of auricular medicine as a tool for diagnosis and therapy. The use of the RAC signal enabled the development of interference field acupuncture as well as the therapeutic so-called “controlled acupuncture”, in which the acupuncture needle is positioned under RAC control and not, as in the classical Chinese tradition, by Cun and others (finger) measurement units [5].

The true nature of RAC, however, remained mysterious despite intense research over many decades.

So far, RAC has been detected manually by placing the therapist’s thumb at the height of the radial pulse of the patient. By developing a “suitable” pressure, it seems to be possible for experienced users to feel a change in the patient’s pulse after triggering stimulation on the ear (and later on the body), which felt as if the pulse beating had increased slightly for one to three beats.

As this method of measurement could not easily be conveyed to many therapists and the explanatory ability in the sense of a physiological derivation was not given, over the years RAC threatened more and more as an objective criterion for a patient response in the sense of a reproducible measurement on the basis of scientificity to lose its authority.

In 2014 Litscher et al. [6] from Austria published together with German and Chinese researchers an original article in Integrative Medicine International. A new high-resolution imaging technique for the registration of pulsatory surface changes might allow RAC to be quantified reproducibly for the first time. That method combines an innovative microscope system (available at the Medical University of Graz), video analysis software, and special image processing software (from the Beijing University of Science and Technology). Even small, pulse-dependent alterations of the skin surface could be clearly visualized. The pilot measurements confirmed the validity of the new methodological approach [6].

Recently in 2017, Moser et al. [7] also from the Medical University of Graz in Austria published proof that RAC follows physiological laws quite well. The study was based on a previous series of tests based on heart rate variability measurements and analyses and showed various reproducible changes of physiological parameters. A total of seven parameters were recorded, as well as different external non-invasive stimuli under a defined set-up and test course. The authors assumed that RAC is a manifestation of a cardiac reaction of the autonomic nervous system, which then—in the sense of a stimulus response—triggers a pulse wave. They concluded that the use of subtle stimuli near the limit of perceptibility should be appropriate to allow repeatable testing of the immediate vagal as well as the delayed sympathetic response. In order to determine the efficiency or the diagnostic value of the test series, further studies are necessary. In particular, the pathways from the source of the stimulus to the autonomic nervous system (ANS) response would vary depending on the stimulus selected and be complex. Individual patient features have not yet been looked at in depth.

Since most of the previous considerations on the physiology of RAC were not completely satisfactory, two authors of this editorial (Dr. Theodoros Yannacopoulos and Dr. Peter Kreisl), started with the development and preparation of the current state of science in various thematic subareas. One goal was to follow the pathway of the stimulus, e.g., from the ear, to the point of reaction at the radial pulse in the sense of a physiological chain of events. The processing of the physiology should then be the basis for being able to develop an apparatus that makes it possible to represent RAC simply, safely, and reproducibly based on physiologically proven findings as mentioned before. This equipment should be able for example, to help untrained users gathering RAC. It could also be used for training purposes. Another use of such an apparatus could also be to serve as a control unit in the sense of a biofeedback mechanism in which a subsequently switched diagnostic and/or therapeutic device (e.g., bioresonance device) only triggers certain subsequent steps, if the RAC response of the patient has been authorized.

The two investigators (T.Y. and P.K.) assume that RAC cannot be interpreted in the sense of a cardiac ANS response with the triggering of a pulse wave. Rather, their hypothesis defines RAC as a purely electrophysiological phenomenon in the sense of a cerebral response and transmission. The purpose of the present small series of measurements is to show that the RAC signal is additive to the RR reaction wave as an electrical signal.

A further purpose of this article is also to demonstrate how an irritation triggered by the ear takes its pathway and then ultimately triggers an RAC reaction. For this reason, the so-called control point of the deep layer at the ear of manual dominance was stimulated with various stimuli at a healthy volunteer. Although not absolutely necessary for the set-up, in the sense of the system of auricular medicine, the person was first tested concerning energetic disturbances (oscillation/inversion) that could have triggered any paradoxical reactions in terms of false positive or negative RAC reactions or none at all (in the presence of a reaction blockade or an electromagnetic reset obstacle). Since RAC is indeed sometimes used within the framework of energy tests, this preliminary examination in the sense of auricular medicine seemed useful, but in the final analysis is not decisive for the set-up of the present procedure.

The control point of the deep layer was addressed without contact (black and white hammer, 3 Volt hammer, using the plus and minus sides, F-hammer according to Bahr, also both sides and magnetic hammer, using the north and south side) [5]. Moreover, it was addressed by direct, short-pulse pressure application—acupuncture point Shenque CV8 (energetic center, navel)—also on the dominant side. In addition to the control point of the deep layer, the nucleus tractus solitarii, which has a strong relationship to the vagus nerve, should be activated. The considerations of the co-authors assumed that due to the anatomical proximity, a stimulus above the so-called control point of the deep layer would respond to the subthalamic nucleus (NST). The same assumption applies to the approach of the nucleus tractus solitarii (NTS) via stimulus release at the back of the head at the level of about C3/C4. This circuit or its disturbance is also responsible for the etiogenesis of Parkinson’s disease. The subthalamic nucleus is functionally one of the basal ganglia, thus projecting onto it. The subthalamic nucleus is also part of the complex motor control: it receives excitatory fiber access directly from the motor cortex, as well as inhibitory impulses from the outer segment of the globus pallidus and sends it mainly to its inner segment and the substantia nigra. It would be interesting in this context to have knowledge about the exact meaning of the zona incerta. However, this is not necessary for the present conclusion. The mechanisms of glia and their specification seem to be of key importance to the co-authors in the genesis of RAC. In particular, these facts serve to substantiate the hypothesis that RAC is not a pressure wave, but exclusively an electrophysiological phenomenon. This is underpinned by the fact that the transmission of stimuli can be very rapid. The Hodgkin–Huxley model for example describes the functioning of neurons close to biological conditions using the example of action potentials in the giant axon of the octopus. It describes the ion mechanism that occurs in the excitement and inhibition in the peripheral and central areas of the nerve cell membrane. Hodgkin and Huxley received the Nobel Prize for their research in 1963 [8]. However some authors explain that this model is not suitable for explaining various phenomena associated with neuronal excitation for example the pattern of heat emission and absorption and the expansion of cell diameter. In fact, mechanical changes during the action potential have been found in various experiments, indicating that the action potential is accompanied by a mechanical pulse and this could be relevant for RAC.

On the basis of the present knowledge of the central glia, the peripheral glia, and myelin sheaths, the description of RAC in the sense of a cardiac pulse wave does not seem to be the only explanation. Rather, it shows that one must consider the nature of RAC differentiated. As mentioned above, action potentials can trigger a mechanical pulse. This is usually very small and therefore not everywhere to grope on the body. Only at the locations where vascular structures making the heart pulse feel are at the surface of the body, does it seem even possible to palpate RAC. For this reason, most acupuncturists who work with RAC also test RAC on the patient’s radial pulse. According to T.Y. and P.K. the decisive momentum for RAC, however, is the fact that the perceived RAC ultimately does not describe a cardiac response of the ANS, as described previously [7]. Rather, the thermodynamic approach shows that the mechanical pulse of the myelin sheath, triggered by the action potential, is superimposed on the actual cardiac pulse wave. 

Interesting in this context are two other research results. On the one hand, Johannes Fleckenstein repeatedly points out in lectures that the acupuncture point Neiguan PC 6 is supposed to correlate with the motoric vagus nucleus (personal communication). On the other hand there is a publication from 2012 by Litscher et al. [9], in which the authors found that a small but reproducible human cerebral evoked potentials after bilateral nonperceptible laser needle (658 nm, 40 mW, 500 μm, 1 Hz) irradiation of the Neiguan acupoint (PC6) can be triggered. The later results which are unique in scientific literature were obtained in a 26-year-old female healthy volunteer in a joint study between the Medical University of Graz, the University of Graz, and the Graz University of Technology. The findings of the 32-channel evoked potential (EP) analysis indicate the exposure to a frequency of 1 Hz can modulate the ascending reticular activating system.

Intuitively, Paul Nogier had already recognized this by saying that one had to learn to feel RAC. The therapist who uses RAC must therefore recognize the fine mechanical pulse of the myelin sheath in addition to the existing pulse wave and differentiate via the sense of touch of her or his thumb. A controlled stimulus transmission as a result of a cerebral response should be the basis for the measurements of RAC described by Yannacopoulos and Kreisl in the following. 

Based on the assumption that RAC must be an electrical signal superimposed on the radial pulse, the idea arose that one should be able to record this with a technical system. The following recordings were made with a “Micro Ambulatory ECG Recorder, Model DiCare-m1CP” from Dimetek Ltd., China. The first measuring electrode was placed at the level of the radial pulse, where RAC is also tested in practice. The second electrode was placed on the inside of the forearm, just below the joint and thus slightly below the acupuncture point Shaohai (He 3).

The measurement was performed on a randomly requested healthy volunteer who agreed to participate in the testing. P.K. carried out the measurement and stimulus release. In order to exclude a mental influence, T.Y. who is very experienced in RAC and also mental testing, left the room.

For the testing procedure a black-white hammer, a magnetic hammer, a classic 3-V hammer, and the newer F-hammer, contactless above the control point of the deep layer, by the hammer was passed in one movement over the point [5]. In addition direct contact of the point CV 8 with the black and white hammer was tested. Figure 1 shows one example of the measurements.

The investigators found that when using the black and white hammer, RAC is weaker in direct contact with the ear than when moving at the control point. The magnet hammer triggers a very clear RAC and RAC with the F-hammer is less powerful. The different reactions on the ± side underline the thesis of a purely electrical phenomenon. 

The specific electrical patterns triggered by the various stimuli can be detected as RAC by a mathematical calculation. The two investigators (P.K. and T.Y.) claim that this could be the basis for a device-specific implementation in an apparatus that can measure RAC.

The first author of this editorial has the opinion that in the future, based on the published results so far, a multimodal concept for RAC research will emerge (Figure 2). It will focus on the non-contact measurement of surface changes [6] as well as the registration of biosignals that reflect the activity of the ANS [7] but also the hypothesis that RAC has a purely electrophysiological correlation as stated in this editorial by the two co-authors should be implemented.

## Figures and Tables

**Figure 1:**
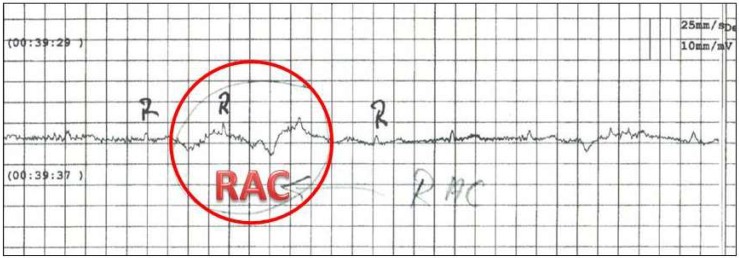
Typical measurement of the micro-electrocardiographic activity. Note RAC is visible as a superimposed signal.

**Figure 2:**
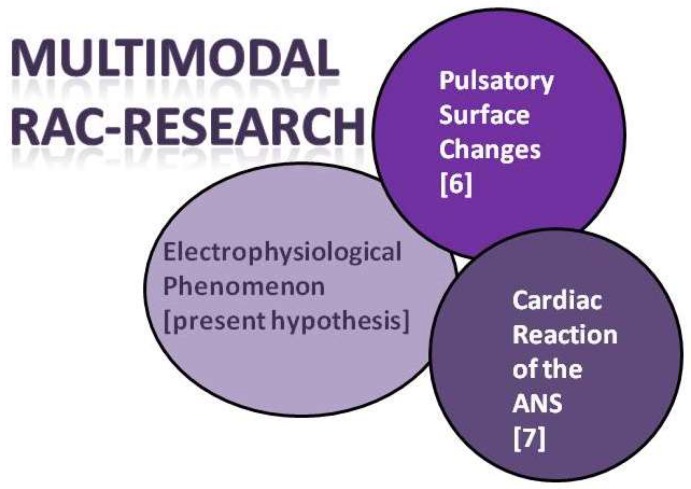
Multimodal RAC research concept for future research.
